# Insights into How Degradable Microplastics Enhance Cu^2+^ Mobility in Soil Through Interfacial Interaction

**DOI:** 10.3390/toxics13090795

**Published:** 2025-09-18

**Authors:** Hongjia Peng, Bolun Yu, Zuhong Lin, Haipu Li

**Affiliations:** 1 Center for Environment and Water Resources, College of Chemistry and Chemical Engineering, Central South University, Changsha 410083, China; 18528037456@163.com (H.P.); 222311089@csu.edu.cn (B.Y.); lzhzzzzgkbs@163.com (Z.L.); 2 Key Laboratory of Hunan Province for Water Environment and Agriculture Product Safety, Changsha 410083, China

**Keywords:** degradable microplastics, soil, Cu^2+^, interfacial behavior, influence mechanism

## Abstract

The incomplete degradation of degradable plastics may pose potential ecological risks, as it can generate degradable microplastics (DMPs), especially when these DMPs coexist with heavy metals in soil. Taking petrochemical-based poly(butylene adipate-co-terephthalate) (PBAT) and bio-based polylactic acid (PLA) as representative DMPs, this study investigated how DMPs affect the adsorption–desorption behavior of Cu^2+^ in soil and the underlying mechanisms via batch equilibrium experiments and characterization analyses. The experiments revealed that ion exchange (accounting for 33.6–34.3%), oxygen-containing functional group complexation, and electrostatic interactions were the primary adsorption driving forces, with chemical adsorption playing the main role. Compared to the soil, the PBAT and PLA had smaller specific surface areas and pore volumes, fewer oxygen-containing functional groups, and especially lacked O-metal functional groups. They can dilute soil, clog its pores, and cover its active sites. 1% DMPs significantly reduced the soil’s equilibrium adsorption capacity (Q_e_) (3.7–4.7%) and increased equilibrium desorption capacity (Q_De_) (1.7–2.6%), thereby increasing the mobility and ecological risk of Cu^2+^. PBAT and PLA had no significant difference in effects on the adsorption, but their specific mechanisms were somewhat distinct. Faced with the prevalent, worsening coexistence of DMPs and heavy metals in soil, these findings contribute to the ecological risk assessment of DMPs.

## 1. Introduction

To solve traditional plastic pollution, scientists have developed degradable plastics such as PBS, PBAT, PLA, and PHA [1,2]. However, these materials require strict conditions (e.g., specific temperature, moisture, oxygen levels, pH, composting duration, and microbial communities) for complete degradation; in natural environments, they still generate DMPs [1,3]. Unlike non-degradable microplastics (NDMPs), DMPs have unique properties [4,5]. For example, DMPs PBS, PBAT, and PLA exhibited distinct microbiome, functionality, and metabolome changes with NDMPs PS, PE, and LDPE in soil [2,6,7]. DMPs PBS and PLA exhibited higher adsorption capacities for the broad-spectrum insecticide fipronil compared with NDMPs PP, PE, PS, and PVC [8]. Additionally, DMPs PBAT and PLA had a more toxic effect on soybean growth than NDMPs LDPE [9]. With the expanding production and application of degradable plastics, the input of DMPs into soil ecosystems is expected to surge.

Mining, smelting, extensive use of industrial products (plastics, pesticides, etc.), sewage irrigation, sludge utilization, and atmospheric transportation settlement have led to a large amount of heavy metals (Cu, Zn, Cd, Cr, Pb, As, etc.) entering the soil, causing serious soil pollution [10]. Among these metals, Cu has received relatively limited attention despite its 2.1% exceedance rate at monitoring sites across China. Cu contaminants mainly accumulate in the top 20 cm of cultivated soil layers, where they disrupt soil physicochemical properties, inhibit soil enzyme activity, alter microbial communities, impair crop yield and quality, and ultimately pose risks to human health via the food chain [11]. The toxicity of Cu depends on its concentration and chemical speciation, which are largely regulated by adsorption–desorption processes—these processes are closely linked to soil organic colloids (humic acid, fulvic acid), inorganic colloids (carbonates, phosphates, clays, metal oxides/hydroxides), and organic-inorganic composite colloids [12,13].

Soil, as the primary sink for MPs, is greatly impacted by MPs, which affect soil organic, inorganic, and organic-inorganic composite colloids. This alters the soil’s adsorption of heavy metals, influencing their mobility and activity [14,15]. For instance, PE can significantly increase the bioavailability of cadmium in soil, exacerbating the absorption and accumulation of cadmium in lettuce [16]; PE and PP MPs could reduce the chemical adsorption of Cd in paddy soil, increasing its bioavailability and bioaccessibility [17]; aged PS could change the bioavailability and leachability of Cd in soil [18]; and pristine PE decreases soil adsorption of As, while pristine and aged PLA enhance As adsorption [19]. These findings collectively confirm that co-occurring MPs and heavy metals can alter metal mobility, raising potential ecological concerns [20]. However, most existing studies focus on the effects of NDMPs on soil heavy metals—a critical knowledge gap remains regarding how DMPs regulate the migration and transformation of heavy metals.

Based on this, this study aims to understand how DMPs affect the migration of Cu^2+^ in soil through interfacial interactions. Two common DMPs (petrochemical-based DMPs: PBAT, and bio-based DMPs: PLA) will be selected to conduct adsorption kinetics, desorption kinetics, and isothermal adsorption experiments and utilize SEM (Scanning electron microscopy), FT-IR (Fourier transform infrared), XPS (X-ray photoelectron spectroscopy), and BET (Bet surface area and porosity) techniques for characterization. By analyzing these experiments, we will identify the types of forces involved in the adsorption processes and evaluate the impact and mechanism of DMPs on soil adsorption behavior. Given the prevalent and increasingly severe issue of DMPs coexisting with heavy metals in soil, this study will clarify how DMPs from different sources affect soil health by altering soil’s ability to retain heavy metals and contribute to a more comprehensive and accurate ecological risk assessment of DMPs.

## 2. Materials and Methods

### 2.1. DMPs and Reagents

Petrochemical-based DMPs PBAT (CAS: 55231-08-8) and bio-based DMPs PLA (CAS: 26100-51-6) were obtained from Dongguan Zhangmutou Plastic Industrial Development Co., Ltd. (Dongguan, China), and their basic physicochemical properties are shown in Table S1. The aforementioned two types of degradable plastics were crushed and sieved through a 60-mesh screen. To wash away latent heavy metals on their surfaces, the DMPs were rinsed with 0.1 mol/L HNO_3_, followed by rinsing with tap water and then with distilled water. Finally, they were dried at 35 °C.

For this study, the chemicals CuSO_4_⋅5H_2_O, NaNO_3_, and HNO_3_ (65% *v*/*v*) were of guaranteed reagent grade and purchased from Shanghai Aladdin Biochemical Technology Co., Ltd. (Shanghai, China). CuSO_4_·5H_2_O was dissolved in distilled water to prepare a 1000 mg/L Cu^2+^ stock solution (pH = 4.20). The desired Cu^2+^ working solution (pH = 4.20) concentration was achieved by diluting the stock solution.

### 2.2. Soil Collection and Incubation

The soil used in this study was collected from Yuelu District, Changsha City, Hunan Province, China (28°10′40″ N, 112°55′27″ E). Surface soil from a depth of 0–20 cm was sampled using the diagonal sampling method. Soil samples from 5 different sampling sites were first homogenized (impurity removal and quartering mixing) before being randomly grouped and air-dried. According to experimental requirements, the soil was sieved through appropriate mesh sizes for soil property analysis and incubation experiments. Detailed physicochemical properties of the soil are provided in Table S2. As can be seen, the soil exhibits low pH, low organic matter content, and weak fertilizer retention capacity, thus qualifying it as a relatively typical acidic red soil in southern China.

In light of relevant literature and the current status of MPs contamination in soil, 1% (*w*/*w*) of DMPs was mixed into the soil samples (<2 mm) [21,22]. An amount of 100 g of mixed soil samples was placed in 250 mL conical flasks and incubated for one month at 25 ± 1.0 °C in a greenhouse. Each sample was weighed at two-day intervals to assess moisture loss, and deionized water was added as needed to maintain soil moisture at 15.9% (*w*/*w*, 60% of field water holding capacity) [17]. Post-incubation, soil samples were air-dried and sieved through a 60-mesh sieve for subsequent experiments.

### 2.3. Adsorption and Desorption Experiments

#### 2.3.1. Adsorption Kinetics Experiment

1.0000 g of soil sample (with/without DMPs) was added to a 50 mL centrifuge tube. Then, 25.00 mL of Cu^2+^ solution (the theoretical concentration was set to 200 mg/L and the pH was 4.20) was added. Additionally, 1.0000 g of soil was weighed, placed in a separate 50 mL centrifuge tube, and 25.00 mL of UP water (pH = 4.20) was added to measure the error caused by the background value of Cu^2+^ in the soil. The centrifuge tubes were placed in a thermostatic shaker (25 ± 1 °C, 150 ± 5 rpm, model TS-200DC, Tensuc, Shanghai, China). Samples were collected at 0, 0.25, 0.5, 1.0, 1.5, 2.0, 3.0, and 4.0 h. The solution was filtered using 0.45 μm filter membranes to analyze the Cu^2+^ concentration, and each sample was measured in triplicate. Three replicates and a soil-free blank treatment were included in each experiment.

For soil samples, their adsorption capacity (Q_e_, Q_t_) and adsorption proportion (A) for Cu^2+^ were determined by the difference between the amount of Cu^2+^ added and that retained in the solution, using (Equations (1)–(3)) [17,23]:

(1) $$Q_{e}={(C}_{0}-C_{e})\times V/M$$

(2) $$Q_{t}={(C}_{0}-C_{t})\times V/M$$

(3) $$A={(C}_{0}-C_{e})/C_{0}\times 100\%$$

where Q_e_ and Q_t_ (mg/g) represent the amounts of Cu^2+^ adsorbed at equilibrium and at time t, respectively; C_0_, C_t_, and C_e_ (mg/L) denote the concentrations of Cu^2+^ in the solution before adsorption, at time t, and at equilibrium, respectively; V (L) is the volume of the solution added, M (g) is the weight of the soil sample, and A (%) is the adsorption proportion.

To investigate the adsorption and desorption kinetics, the pseudo-first-order (PFO) model (Equation (4)), pseudo-second-order (PSO) model (Equation (5)), and intraparticle diffusion (ID) model (Equation (6)) were applied [24].

(4) $$Q_{t}=Q_{e}(1-\exp(-k_{1}t))$$

(5) $$\frac{t}{Q_{t}}=\frac{t}{Q_{e}}+\frac{1}{k_{2}Q_{e}^{2}}$$

(6) $$Q_{t}=K_{i}\times t^{0.5}+C$$

where k_1_ (h^−1^) and k_2_ (g (mg·h)^−1^) represent the equilibrium rate constants for the PFO and PSO models, respectively; t is the contact time (h), K_i_ (g·mg^−1^ h^−0.5^) is the rate constant of the ID model, and C is the boundary layer diffusion constant.

#### 2.3.2. Desorption Kinetics Experiment

Based on previous studies [13], once the adsorption process was completed, the centrifuge tube was removed, centrifuged, and the supernatant was poured out. For the desorption experiments, 25.00 mL of 0.1 mol/L NaNO_3_ (pH = 4.20) was added to each tube. Samples were collected at 0, 0.25, 0.5, 1.0, 1.5, 2.0, 3.0, and 4.0 h. The solutions were filtered through 0.45-μm filter membranes to determine the Cu^2+^ concentration.

The desorbed Cu^2+^ (Q_De_, mg/g) and desorption proportion (A_De_, %) were calculated using (Equation (7)) and (Equation (8)), respectively:

(7) $$Q_{\mathrm{De}}=(C_{\mathrm{De}}\times (25+W_{2}-W_{1})-C_{e}\times (W_{2}-W_{1}))/\left(1000M\right)$$

(8) $$A_{\mathrm{De}}=Q_{\mathrm{De}}/Q_{e}\times 100\%$$

where C_De_ (mg/L) represents the concentration of Cu^2+^ in the solution after desorption, W_1_ (g) denotes the weight of the tube and soil sample, and W_2_ (g) represents the weight of the tube, soil sample, and post-adsorption residual solution.

#### 2.3.3. Isotherm Adsorption Experiment

The isothermal adsorption experiments were carried out under the same conditions as the adsorption kinetics experiments. The theoretical concentrations of Cu^2+^ were established at 100, 150, 200, 250, and 300 mg/L, and the pH was 4.20. The adsorption equilibrium time was set to 2 h. After reaching adsorption equilibrium, the centrifuge tubes were removed and centrifuged, and the supernatant was collected to determine the Cu^2+^ concentration. In the experimental group where the initial Cu^2+^ concentration was set at 200 mg/L, the concentrations of Cu^2+^, Mg^2+^, Al^3+^, K^+^, Ca^2+^, Mn^2+^, and pH were measured after equilibrium was achieved.

P_ie_ was the proportion of Cu^2+^ adsorption attributed to ion exchange and calculated using (Equations (9)–(11)) [25]:

(9) $$D_{e}=C_{0}-C_{e}$$

(10) $$C(H^{+})=10^{\wedge }(-\mathrm{pH})$$

(11) $$P_{\mathrm{ie}}=(2D_{e}({\mathrm{Mg}}^{2+})+3D_{e}{(\mathrm{Al}}^{3+})+D_{e}(K^{+})+2D_{e}({\mathrm{Ca}}^{2+})+2D_{e}({\mathrm{Mn}}^{2+})+D_{e}(H^{+}))/2D_{e}({\mathrm{Cu}}^{2+})$$

where D_e_ (mg/L) refers to the difference in ion concentrations before and after reaching adsorption equilibrium. C(H^+^) (mg/L) denotes the concentration of H^+^.

The partition coefficient (K_d_ (L/g)) was determined using (Equation (12)):

(12) $$K_{d}=Q_{e}/C_{e}$$

The Freundlich (Equation (13)), Langmuir (Equation (14)), and Dubinin–Radushkevich (D-R) (Equation (16)) models were employed to investigate the isotherm adsorption experiments.

(13) $$Q_{e}=K_{f}\times C_{e}^{1/n}$$

where K_f (_mg·g^−1^/(mg·L^−1^)^1/n^) represents the Freundlich constant. The 1/n (dimensionless) represents the Freundlich intensity, indicating the strength of the adsorption driving force or the surface heterogeneity. The value of 1/n reflects the difficulty of adsorption: generally, a value of 0 < 1/n < 1 suggests an easily adsorbed process; when 1/n > 1, it indicates a difficult adsorption process; and when 1/n = 1, it denotes an irreversible process [26].

(14) $$Q_{e}=Q_{m}\times b\times C_{e}/(1+b\times C_{e})$$

where Q_m_ (mg/g) represents the maximum absorption capacity, and b (L/mg) denotes the affinity constant.

The separation factor (R_L_) was calculated according to (Equation (15)):

(15) $$R_{L}=1/(1+b\times C_{0})$$

where R_L_ can be used to identify the type of adsorption: when R_L_ > 1, it indicates that adsorption is unfavorable, categorized as IUPAC Type III adsorption; when R_L_ = 1, it signifies linear adsorption; when 0 < R_L_ < 1, it indicates favorable adsorption, classified as IUPAC Type I adsorption; and when R_L_ = 0, it denotes irreversible adsorption [26,27].

The adsorption free energy (E (J/mol)) was calculated according to (Equations (16)–(18)):

(16) $$\ln Q_{e}=\ln Q_{m}-\beta \varepsilon^{2}$$

(17) $$\varepsilon =\mathrm{RTln}(1+\frac{1}{C_{e}})$$

(18) $$E=\frac{1}{\sqrt{2\beta }}$$

where β (mol^2^/kJ^2^) is a parameter associated with the mean adsorption energy. ε (kJ·mol^−1^) (Equation (17)) represents the Polanyi potential energy, which is related to equilibrium concentration. R (8.314 J/(mol·K)) represents the gas constant. T (K) denotes the absolute temperature. When E < 8 kJ·mol^−1^, it indicates physical adsorption; when E > 8 kJ·mol^−1^, it indicates chemical adsorption [28,29].

### 2.4. Characterization and Determination

The points of zero charge (pH_pzc_) for surfaces of soil and DMPs were determined using the pH drift method [30]. The specific surface area and pore distribution of soil and DMPs were measured using an automated specific surface area and porosity analyzer (BET, Micromeritics ASAP 2460, Norcross, GA, USA). High-magnification images of DMPs and soil were characterized using a scanning electron microscope (SEM, JEOL, Tokyo, Japan). The surface functional groups of the samples were recorded by Fourier transform infrared spectroscopy (FT-IR; Nicolet iS50, Thermo Fisher Scientific, Waltham, MA, USA). The elemental composition and chemical bonds responsible for Cu^2+^ adsorption in the samples were characterized by X-ray photoelectron spectroscopy (XPS; Thermo Scientific ESCALAB Xi+, Waltham, MA, USA). The concentrations of Cu^2+^ and other metal ions were measured by inductively coupled plasma optical emission spectrometry (ICP-OES, Optima 8000, PerkinElmer, Waltham, MA, USA).

### 2.5. Quality Control and Data Analysis

The experimental data from three replicates were averaged, and graphs were plotted with Origin 2019. All statistical analyses were performed using SPSS 26.0. The statistical significance of differences between the treatment groups (n = 3) and the control group was determined by one-way analysis of variance (ANOVA) and Duncan’s test, with significance set at *p* < 0.05.

## 3. Results and Discussion

### 3.1. Adsorption Kinetics Experiment Analysis

#### 3.1.1. Effect of Contact Time on the Adsorption Process

**Figure 1:**
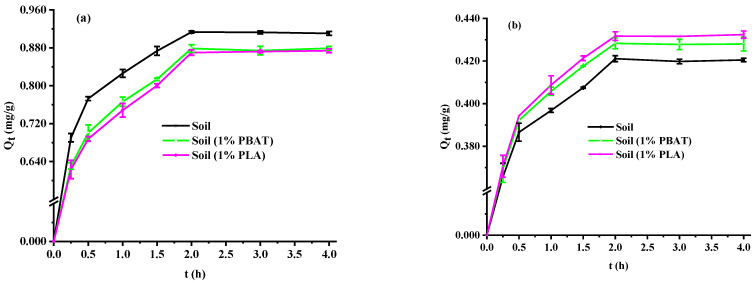
Effects of contact time on Cu^2+^ adsorption (**a**) and desorption (**b**).

As shown in Figure 1a, the adsorption process of Cu^2+^ could be divided into three main stages: (1) the rapid adsorption phase, within 0.25 h, the adsorption reached 71.6–75.6% of Q_e_; (2) the slow adsorption phase from 0.25 to 2.0 h; and (3) the equilibrium phase after 2.0 h. This phenomenon can be attributed to the gradual reduction in the Cu^2+^ concentration gradient between the aqueous solution and the soil adsorbent surface, as well as the high active sites on the surface of the adsorbent being gradually occupied [17,31]. This indicates that the adsorption of Cu^2+^ is a complex heterogeneous diffusion process characterized by an exponential decrease in the adsorption rate with increasing contact time and adsorption capacity [32]. The Q_e_ under different treatments, from largest to smallest, was as follows: soil > soil (1% PBAT) > soil (1% PLA). Among them, the difference between soil (1% PBAT) and soil (1% PLA) was not significant. This indicates that the 1% DMPs reduced Q_e_ (3.7–4.7%), respectively. Because the soil has a complex composition containing various minerals and organic matter, whereas the surface properties of the two DMPs are relatively simple [17,33,34]. The two types of DMPs have a much weaker adsorption capacity for Cu^2+^ compared to soil. Chen et al. [35] also found that the adsorption capacity of sediment for Cu^2+^ was significantly higher than that of both pristine and aged MPs. In addition, the DMPs may also block soil pores and cover active sites, thereby reducing the adsorption of Cu^2+^ [36]. The previous research found the soil (1% PS) significantly reduced Q_e_ by 8.0% compared to soil [25]. This indicates that the reduction in soil adsorption capacity caused by the two DMPs is less than that caused by the equivalent amount of PS NDMPs. The possible reasons are the different physicochemical properties of the two DMPs in comparison with PS MPs [1,37].

#### 3.1.2. Adsorption Kinetic Model Fitting

As shown in Figure S1 and Table 1, the adsorption kinetics of Cu^2+^ were fitted with the PFO, PSO, and ID models. By comparing R^2^, Q_e_,^cal^, and Q_e_,^exp^, it could be observed that the fitting performance of the PSO model was superior to that of the PFO and ID models for describing the adsorption kinetics. This indicates that the adsorption rate is predominantly governed by its adsorption onto the active sites of the soil adsorbent, with partial influence from the diffusion steps. In the PFO and PSO models, the reaction rate constants K_1_ and K_2_ exhibited the same trend as Q_e_,^exp^; that is, the smaller the Q_e_,^exp^, the smaller the values of the reaction rate constants. The DMPs reduced K_1_ and K_2_, likely due to the blocking effect of DMPs, which hinder the diffusion and adsorption of Cu^2+^ onto soil particles [34,36]. In the ID model, R^2^ were all greater than 0.6, indicating that this model also fits the adsorption kinetics process well. The boundary layer diffusion constant C also varied consistently with Q_e_,^exp^; that is, the smaller the Q_e_,^exp^, the smaller the C. This suggests that the DMPs reduced the thickness of the diffusion boundary layer, weakening the boundary diffusion effect while strengthening the intraparticle diffusion effect [17].

In short, the adsorption process of Cu^2+^ on different soil samples included three stages: rapid, slow, and equilibrium phases. 1% DMPs reduced Q_e_ (3.7–4.7%) due to their dilution, blocking, and covering effects. PSO models best described these processes, with DMPs reducing adsorption rate because of their blocking effect.

### 3.2. Desorption Kinetics Experiment Analysis

#### 3.2.1. Effect of Contact Time on the Desorption Process

As shown in Figure 1b, the desorption process could also be categorized into three primary stages: (1) During the initial 0.25 h, the desorption rate was rapid, with the amount desorbed reaching 85.8–86.8% of Q_De_. (2) From 0.25 to 2.0 h, the desorption rate gradually slowed down. (3) After 2.0 h, desorption equilibrium was reached. This rapid desorption at the beginning is attributed to the relatively high Cu^2+^ content in the soil samples and the significant concentration difference between the soil and the desorption solution. As the desorption time increases, the Cu^2+^ content in the soil samples diminishes, leading to a reduced concentration difference and a slower desorption rate [25,34]. This indicates that the desorption of Cu^2+^ is also a complex heterogeneous diffusion process, characterized by an exponential decrease in the desorption rate with increasing contact time and desorption capacity [32]. The Q_De_ for different treatments, ranked from smallest to largest, were as follows: soil < soil (1% PBAT) < soil (1% PLA), Among them, the difference between soil (1% PBAT) and soil (1% PLA) was not significant. This indicates that the 1% DMPs increased Q_De_ (1.7–2.6%), respectively. The desorption rates were as follows: soil (46.1%) < soil (1% PBAT, 48.7%) < soil (1% PLA, 49.7%). This suggests that the treatment group with the highest Cu^2+^ adsorption had the lowest desorption rate, while the addition of DMPs enhanced the desorption rate of Cu^2+^. Because the adsorption strength of Cu^2+^ on DMPs is significantly weaker than that in the soil, allowing Cu^2+^ adsorbed on the surface of DMPs to be easily desorbed [31,35]. Additionally, the coverage of highly active sites in the soil by DMPs forces some Cu^2+^ to adsorb onto low-activity sites instead, making them more prone to desorption.

#### 3.2.2. Desorption Kinetic Model Fitting

As shown in Figure S1 and Table 1, the desorption kinetics of Cu^2+^ were fitted with the PFO, PSO, and ID models. Based on R^2^ and the comparison between Q_De_,^cal^ and Q_De_,^exp^, it was found that the PSO models provided a better fit than the PFO models, whereas the ID models fitted poorly. Among the different treatment groups, the desorption rate constants K_1_ and K_2_ decreased as Q_De_,^exp^ increased. This indicates that DMPs reduced the desorption rate of Cu^2+^, likely due to the blocking effect of DMPs, which hinder the desorption of Cu^2+^ by NaNO_3_ [38].

In short, the desorption process of Cu^2+^ on different soil samples included three stages: rapid, slow, and equilibrium phases. 1% DMPs increased Q_De_ (1.7–2.6%), due to their dilution, blocking, and covering effects. PSO models best described these processes, with DMPs reducing desorption rates because of their blocking effect.

### 3.3. Isotherm Adsorption Experiment Analysis

#### 3.3.1. Effect of Initial Concentration on the Adsorption Process

**Figure 2:**
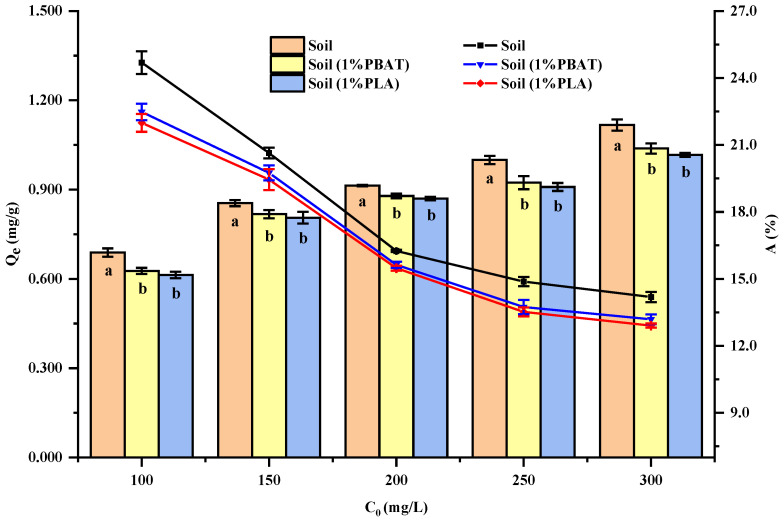
Effect of initial Cu^2+^ concentration on the adsorption of soil samples with different DMPs.

When adsorption reaches equilibrium at a specified temperature, the isotherm adsorption can illustrate the distribution of the adsorbate between the solid and liquid phases [17,39]. According to Figure 2, the isothermal adsorption experiments showed that the Q_e_ varied with the different initial Cu^2+^ concentrations. For the same initial Cu^2+^ concentration, the Q_e_ was ranked as follows: soil > soil (1% PBAT) > soil (1% PLA). Among them, the difference between soil (1% PBAT) and soil (1% PLA) was not significant. This trend was consistent with the results of the adsorption kinetics, indicating that the DMPs reduced the soil’s adsorption capacity. As the initial Cu^2+^ concentration increased, the Q_e_ of the same soil sample also rose, whereas adsorption percentage (A) declined. Because soil samples are heterogeneous, they contain both high-affinity and low-affinity adsorption sites. At lower Cu^2+^ concentrations, Cu^2+^ tends to be adsorbed onto high-affinity sites, primarily through complexation. As the concentration increases, these high-affinity sites become saturated, and the excess Cu^2+^ is adsorbed onto low-affinity sites, primarily through ion exchange, electrostatic interactions, and other forces [13,40].

According to Table S3, for the same soil sample, the partition coefficient (K_d_) decreased as the initial Cu^2+^ concentration increased. This is because the adsorption sites on the soil sample surface are limited, and the increase in adsorption quantity slows down gradually [13]. For the same initial Cu^2+^ concentration, soil samples with a larger Q_e_ (soil > soil (1% PBAT) > soil (1% PLA)) exhibited a higher K_d_. This further indicates that DMPs reduced the soil’s adsorption capacity.

#### 3.3.2. Isothermal Adsorption Model Fitting

As shown in Figure S2 and Table 2, isothermal adsorption experiments were fitted with Freundlich, Langmuir, and D-R models. The Freundlich model is suitable for cases where the functional active sites and groups on the surface of the adsorbent are unevenly distributed [41,42]. The Langmuir model is suitable for describing monolayer adsorption on a uniform surface where interactions between molecules at adjacent sites can be neglected [26]. The D-R model is suitable for heterogeneous surfaces and microporous adsorption [42]. In the study, the Freundlich models (0.936 ≤ R^2^ ≤ 0.957) and Langmuir models (0.931 ≤ R^2^ ≤ 0.947) fitted well to the isothermal adsorption, consistent with prior research [43]. This is because the adsorption process may involve multiple mechanisms [43,44]. That is to say, the monolayer adsorption process of Cu^2+^ begins on the outer surface of the soil sample. Then, Cu^2+^ diffuses into the cracks, pores, and interior of the soil, exhibiting multilayer adsorption. Finally, as the concentration of Cu^2+^ increases, the adsorption amount of Cu^2+^ does not significantly increase because the active adsorption sites become progressively occupied [43]. Specifically, the Freundlich model best described the adsorption of Cu^2+^ by soil, and the Langmuir model best described the adsorption of Cu^2+^ by soil (1% PBAT) and soil (1% PLA). Because the complex and heterogeneous composition of the soil results in an uneven distribution of functional active sites and groups. The intervention of DMPs can alter the uniformity of soil and the availability of active sites and groups. The Freundlich model calculated 0 < 1/n < 1, indicating favorable adsorption [26]. The Langmuir model calculated 0 < R_L_ < 1 (Table S4), indicating that adsorption is easy and belongs to IUPAC type I adsorption [26]. Although the D-R models (0.824 < R^2^ < 0.916) performed relatively poorly, it was still acceptable. E > 8 kJ/mol from D-R model fitting, indicating predominantly chemical adsorption [45].

According to Table S5, ion exchange served as a key mechanism for Cu^2+^ adsorption by different soil samples. The ion concentration difference in solution before and after adsorption was Al^3+^ > Ca^2+^ > K^+^ > Mn^2+^ > Mg^2+^ > H^+^. According to the valence conservation law, the contribution of different cations to Cu^2+^ adsorption was also in the order of Al^3+^ > Ca^2+^ > K^+^ > Mn^2+^ > Mg^2+^ > H^+^. The P_ie_ values of different soil samples were soil < soil (1% PBAT) < soil (1% PLA), which was exactly the opposite of the Q_e_, indicating that the DMPs increased the proportion of ion exchange in the soil’s Cu^2+^ adsorption. Ion exchange is an outer-sphere complexation form where there are only weak covalent bonds between the metal and the charged soil surface, making it essentially reversible [33]. This indirectly indicates that DMPs reduced the interaction force between soil particles and Cu^2+^.

In brief, PBAT and PLA DMPs reduced the Q_e_, with the Q_e_ order being soil > soil (1% PBAT) > soil (1% PLA). Among them, the difference between soil (1% PBAT) and soil (1% PLA) was not significant. Higher initial Cu^2+^ concentrations increased overall adsorption but reduced the percentage of adsorbed Cu^2+^. The adsorption process was mainly chemical adsorption, and ion exchange was one of the important adsorption mechanisms. DMPs increased the proportion of ion exchange in the soil’s Cu^2+^ adsorption but reduced the interaction force between soil particles and Cu^2+^.

### 3.4. Mechanisms of Enhanced Cu^2+^ Mobility in Soil by DMPs

The interfacial behavior between soil and Cu^2+^ is influenced by various factors, including soil minerals, organic matter, and environmental conditions [46]. When DMPs entered the soil, they might alter these factors, subsequently affecting the interfacial behavior between soil and Cu^2+^. Therefore, this study conducted a series of characterizations on the samples before and after adsorption to clarify how DMPs influenced the interfacial behavior between soil and Cu^2+^ and to elucidate the specific mechanisms involved.

#### 3.4.1. pH_PZC_ of Soil and DMPs

Electrostatic interaction is among the most common adsorption mechanisms. Soil particles carry a large quantity of negative charge on their surfaces, which can adsorb Cu^2+^ through electrostatic interactions. The pH_PZC_ of the two DMPs and the pH of the soil solution influence the charge on the surfaces of the DMPs. When the pH_PZC_ is lower than the solution pH, the surface of the DMPs is negatively charged. When the pH_PZC_ is higher than the solution pH, the surface of DMPs is positively charged [37,47].

In this study, the pH_PZC_ values for soil, PBAT, and PLA were determined and are shown in Table S6, with values of 2.49, 4.30, and 3.38, respectively. Therefore, when the pH of the solution was 4.20, the surfaces of the soil and PLA were negatively charged, which favored the adsorption of Cu^2+^ through electrostatic interactions. After one month of aging and cultivation, the surface of PLA adsorbed some cations from the soil, allowing PLA to also adsorb Cu^2+^ through ion exchange [48]. On the other hand, the surfaces of PBAT carried a small amount of positive charge, which caused slight electrostatic repulsion. Meanwhile, the PBAT could also adsorb Cu^2+^ through ion exchange. Overall, the electrostatic interactions were relatively weak, contributing only a minor fraction to the total Cu^2+^ adsorption.

#### 3.4.2. SEM Images of the Surface Microstructures of Soil and DMPs

SEM is a high-resolution surface analysis technique that enables direct observation of a material’s microstructure, providing essential morphological data for surface characterization studies [17,35]. The SEM images of soil and the two DMPs are shown in Figure S3. The surface of soil particles was highly uneven and contained abundant pores, indicating their large specific surface area and well-developed porous structure. The PBAT featured many pits and indentations, forming a honeycomb shape. The PLA surface was irregular, with some cracks and pits. The grooves, pits, and pores on the surfaces of soil and DMPs enhance Cu^2+^ adsorption through multiple mechanisms. Their rough texture significantly increases the specific surface area [35,43]. Additionally, the edges of these structures often expose highly reactive chemical sites (e.g., unsaturated bonds, functional groups), facilitating complexation adsorption with Cu^2+^ [17]. Furthermore, the unique topological features regulated fluid dynamics, prolonging the contact time between Cu^2+^ and the soil/DMP surface.

#### 3.4.3. BET Characterization of Soil and DMPs

To better reveal the surface physical properties of soil and DMPs, BET characterization was employed (Table S7). The specific surface area ranked as soil > PLA > PBAT, the pore volume as soil > PBAT > PLA, and the pore diameter as soil > PBAT > PLA. The specific surface area and pore volume of soil particles were significantly larger than those of the two DMPs. This can partly explain why the two DMPs reduced the soil’s adsorption of Cu^2+^ [35]. Because specific surface area and pore volume reflect the extent of contact between soil, DMPs, and Cu^2+^ to some extent [43]. This indicates that specific surface area and pore volume are important factors in determining how DMPs influence the soil’s Cu^2+^ adsorption capacity.

#### 3.4.4. FTIR Characterizations of Soil and DMPs

**Figure 3:**
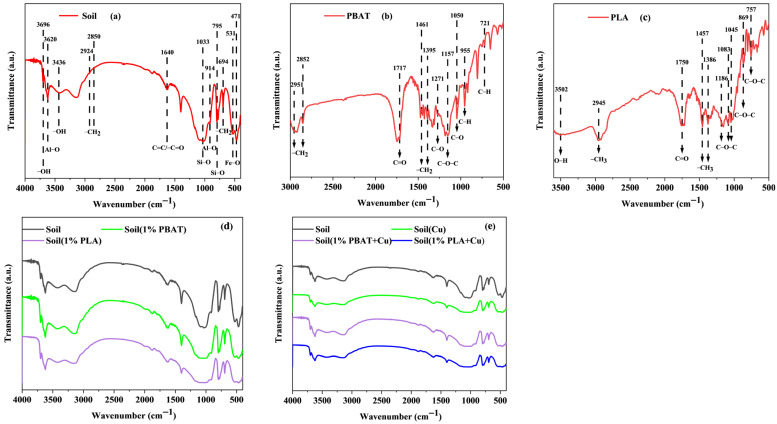
FTIR characterizations of soil (**a**), PBAT (**b**), PLA (**c**), the soil samples after cultivation (**d**), and the soil samples after adsorption (**e**).

The presence of surface functional groups is crucial for adsorption, as they can engage in complexation reactions with Cu^2+^ [18,33]. The functional groups of the soil and the two types of DMPs were characterized using FTIR. As shown in Figure 3, the soil primarily exhibited Fe−O vibrations (471 and 535 cm^−1^), −CH_2_ vibrations (694 cm^−1^), Si−O vibrations (795 and 1033 cm^−1^), Al−O vibrations (914 and 3620 cm^−1^), C=C/C=O vibrations (1640 cm^−1^), −CH_2_ symmetric and asymmetric stretching vibrations (2850 and 2924 cm^−1^), and −OH bending vibrations (3436 and 3696 cm^−1^) [17,25]. The PBAT primarily exhibited out-of-plane C−H bending vibrations on the benzene ring (721 and 955 cm^−1^), C−O stretching vibrations (1050 and 1271 cm^−1^), C−O−C stretching vibrations (1157 cm^−1^), −CH_2_ bending vibrations (1395 and 1461 cm^−1^), C=O stretching vibrations (1717 cm^−1^), and −CH_2_ stretching vibrations (2852 and 2951 cm^−1^) [49,50,51,52]. The PLA primarily exhibited C−O−C out-of-plane bending vibration (869 cm^−1^), C−O−C stretching vibrations (757, 1045, 1083, and 1186 cm^−1^), symmetric −CH_3_ bending vibrations (1386 cm^−1^), asymmetric −CH_3_ bending vibrations (1457 cm^−1^), C=O stretching vibrations (1750 cm^−1^), asymmetric −CH_3_ stretching vibrations (2945 cm^−1^), and −OH stretching vibrations (3502 cm^−1^) [49,52,53]. Based on Figure 3d,e, it can be observed that the FTIR spectra of different soil samples after incubation aging and after adsorption did not show significant differences. This may be because the DMPs and adsorbed Cu^2+^ only covered a small portion of the soil surface, making it difficult to detect these changes with FTIR.

Based on the above observations, it can be seen that in addition to alkyl groups, the soil also contained a significant amount of oxygen-containing functional groups, specifically Fe−O, Al−O, and Si−O functional groups, which were not found in the two types of DMPs. The two types of DMPs mainly consisted of alkyl groups and fewer oxygen-containing functional groups, with differences primarily in their wavenumber and quantity. The differences in functional groups might be one reason why DMPs reduced soil adsorption capacity, as oxygen-containing functional groups (including Fe−O, Al−O, and Si−O) can immobilize Cu^2+^ through surface adsorption and complexation [17,35]. Compared to the previous research, we can find that PS MPs, which primarily consisted of alkyl groups, reduced soil adsorption capacity to a greater extent than the two types of DMPs [25]. This is because the two types of DMPs contain a certain number of oxygen-containing functional groups that can adsorb Cu^2+^ through complexation and are more hydrophilic than PS MPs, resulting in less blockage and coverage of the soil [54]. Additionally, the two types of DMPs may increase the content of dissolved organic carbon in the soil, which can also enhance the interaction between the soil and Cu^2+^ [55,56].

#### 3.4.5. XPS Characterizations of Soil and DMPs

The elemental composition and content of the soil, two DMPs, and adsorbed soil samples were analyzed using XPS technology, as shown in Table 3. The full spectrum analysis revealed that the soil mainly consists of C, O, Si, Al, and K. The two types of DMPs are primarily composed of C and O, with PLA DMPs showing small amounts of Si and Mg. The adsorbed soil samples mainly consist of C, O, Si, Al, and K, with a small amount of Cu detected. However, the atomic ratio of Cu does not show any significant difference, likely because the amount of adsorbed Cu^2+^ was relatively small and XPS full spectrum analysis can only roughly estimate the atomic ratios of different elements [57].

Oxygen-containing functional group complexation is a key mechanism for soil adsorption of heavy metals [35,58]. According to Table 3, the atomic percentages of C1s were PBAT > PLA > soil > soil (1% PLA + Cu) > soil (1% PBAT + Cu) > soil (Cu). The atomic percentages of O1s were soil (Cu) > soil (1% PBAT + Cu) > soil (1% PLA + Cu) > soil > PBAT > PLA. The ratios of O/C were soil (Cu) > soil (1% PBAT + Cu) > soil (1% PLA + Cu) > soil > PBAT > PLA. The atomic percentages of O1s in soil and the O/C ratio of soil were significantly greater than those of the two DMPs, indicating that the soil’s adsorption of Cu^2+^ was much greater than that of the two DMPs, and the DMPs reduced the soil’s Cu^2+^ adsorption through a “dilution effect” [36]. The increase in the atomic percentage of O1s and the O/C ratio in the soil after adsorption also indicates the significant role of oxygen-containing functional groups [35]. The effects of PBAT and PLA DMPs on Cu^2+^ adsorption in soil were not significantly different. The possible reasons are the adsorption of Cu^2+^ by soil samples involves not only complexation with oxygen-containing functional groups but also ion exchange and electrostatic interactions [35,58]. The different surface physicochemical properties of the two DMPs influence these adsorption forces, and the overall impact is not significantly different.

**Figure 4:**
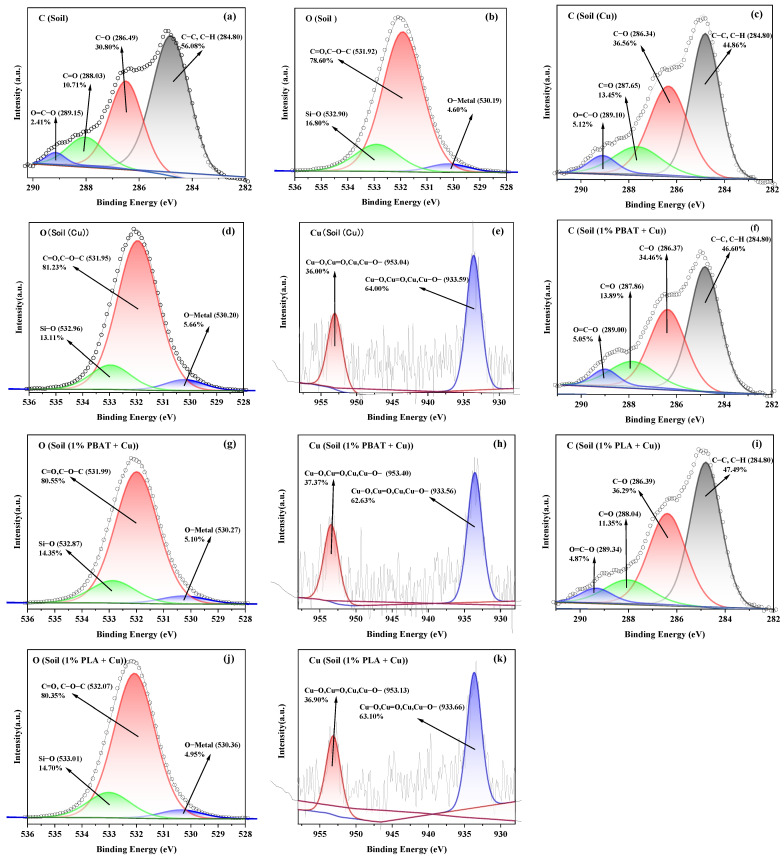
XPS fine spectra of soil before adsorption (**a**,**b**), after adsorption (**c**–**e**), of soil (1% PBAT) after adsorption (**f**–**h**), and of soil (1% PLA) after adsorption (**i**–**k**).

From the XPS fine spectra analysis in Figure S4, it can be seen that the two types of DMPs contain some oxygen-containing functional groups, such as C−O, C=O, and O=C−O, in addition to C−C. Therefore, they can complex with Cu^2+^ [43]. The previous research found that traditional PS MPs almost did not contain oxygen-containing functional groups, which explains why soil samples containing DMPs have a higher adsorption capacity for Cu^2+^ compared to those containing PS MPs [25,59]. After different soil samples adsorbed Cu^2+^ (Figure 4), the C−O, C=O, O=C−O, O−Metal, and C−O−C functional groups increased compared to soil before adsorption, indicating that these functional groups are crucial for the adsorption of Cu^2+^ [39,60]. This is because the oxygen atoms on these functional groups can provide lone pairs of electrons for Cu^2+^ [23,58,61].

In summary, electrostatic interactions, along with complexation by oxygen-containing functional groups, were also key adsorption mechanisms of Cu^2+^. Compared to the soil, the DMPs had smaller specific surface areas and pore volumes, fewer oxygen-containing functional groups, and especially lacked O-metal functional groups. Additionally, they blocked soil pores and covered active soil sites, thereby reducing Cu^2+^ adsorption and increasing desorption.

## 4. Limitations and Future Directions

Despite providing the aforementioned insights, the present study has certain limitations. In this experiment, the incubation time of soil samples was only 1 month, which can only reflect the short-term effects of 1% DMPs on the adsorption of copper ions by red soil. Future studies should investigate the long-term effects of DMPs within a wider concentration range on the adsorption of heavy metals by different soil types. In addition, after PBAT and PLA enter the soil, following a one-month incubation period, they might undergo slight aging and degradation—while a small amount of biofilm might also form on their surfaces. This may exert a certain impact on the adsorption process, which this study failed to adequately explain. Future research should strengthen exploration in this area.

## 5. Conclusions

To address the prevalent and worsening issue of DMPs coexisting with heavy metals in soil, this study explored the effects and mechanisms of two typical DMPs (PBAT and PLA) on soil Cu^2+^ adsorption–desorption. The results show that 1% DMPs significantly reduced soil’s Q_e_ (3.7–4.7%) and increased Q_De_ (1.7–2.6%), with DMP-induced blocking lowering Cu^2+^ adsorption/desorption rates; isothermal adsorption confirmed chemical adsorption as the dominant process (ion exchange contributing 33.6–34.3%); and characterization identified electrostatic interactions and oxygen-containing functional group complexation as additional major mechanisms. Compared to soil, PBAT and PLA had smaller specific surface areas, pore volumes, and fewer oxygen-containing functional groups (and no O-metal groups), exerting dilution, clogging, and coverage effects—though their overall impacts on Cu^2+^ adsorption were not significantly different. Collectively, DMPs promote Cu^2+^ migration in soil via these interfacial interactions, may exacerbate soil pollution, and threaten ecosystem health. This study clarifies the regulatory mechanism of DMPs on soil Cu^2+^ adsorption–desorption and provides a scientific basis for ecological risk assessment of DMPs in soils.

## Figures and Tables

**Table 1:** Kinetic parameters of Cu^2+^ adsorption and desorption by soil samples obtained from the PFO, PSO, and ID models.

**Adsorption**										
**Soil Type**	**Q_e_,^exp^ (mg/g)**	**PFO Model**			**PSO Model**			**ID Model**		
		**Q_e_,^cal^ (mg/g)**	**K_1_ (h^−1^)**	**R^2^**	**Q_e_,^cal^ (mg/g)**	**K_2_ [g·(mg·h)^−1^]**	**R^2^**	**C**	**K_i_ (g·mg^−1^·h^−0.5^)**	**R^2^**
Soil	0.913	0.883	5.510	0.984	0.933	11.308	0.997	0.332	0.379	0.601
Soil (1% PBAT)	0.879	0.839	4.794	0.971	0.898	9.171	0.993	0.291	0.375	0.660
Soil (1% PLA)	0.870	0.830	4.727	0.965	0.891	8.908	0.990	0.283	0.375	0.674
**Desorption**										
**Soil Type**	**Q_De_,^exp^ (mg/g)**	**PFO Model**			**PSO Model**			**ID Model**		
		**Q_De_,^cal^ (mg/g)**	**K_1_ (h^−1^)**	**R^2^**	**Q_De_,^cal^ (mg/g)**	**K_2_ [g·(mg·h)^−1^]**	**R^2^**	**C**	**K_i_ (g·mg^−1^·h^−0.5^)**	**R^2^**
Soil	0.421	0.410	8.487	0.993	0.423	56.091	0.999	0.179	0.162	0.480
Soil (1% PBAT)	0.428	0.419	8.161	0.994	0.432	51.166	0.999	0.181	0.166	0.485
Soil (1% PLA)	0.432	0.422	7.999	0.993	0.436	48.510	0.999	0.181	0.168	0.491

**Table 2:** Langmuir, Freundlich, and Dubinin-Radushkevich (D-R) model parameters for Cu^2+^ sorption by soil samples with or without DMPs.

Soil Type	Freundlich			Langmuir			D-R		
	K_f_ (mg^1−n^·L^n^·g^−1^)	1/n	R^2^	b (L/mg)	Q_m_ (mg/g)	R^2^	E (J/mol)	Q_m_ (mg/g)	R^2^
Soil	0.125	0.387	0.957	1.04 × 10^−2^	1.454	0.931	29.596	1.082	0.824
Soil (1% PBAT)	0.113	0.389	0.937	1.00 × 10^−2^	1.372	0.941	28.198	1.024	0.897
Soil (1% PLA)	0.115	0.390	0.936	0.99 × 10^−2^	1.353	0.947	27.896	1.009	0.916

**Table 3:** Elemental atomic percentages (%) on sample particle surfaces.

Sample Particle	C1s	O1s	Si2p	Al2p	K2p	Cu2p	Mg1s	N1s	S2p	Cl2p	O/C
Soil	17.62	65.67	9.28	6.26	0.40						3.73
PBAT MPs	72.21	27.79									0.38
PLA MPs	71.39	25.25	1.77				0.98				0.35
Soil (Cu)	9.70	72.36	9.74	6.64	0.44	0.11					7.46
Soil (1% PBAT + Cu)	10.99	71.51	9.47	6.57	0.52	0.11					6.51
Soil (1% PLA + Cu)	12.40	70.04	9.58	6.53	0.55	0.11					5.65

## Data Availability

The data that support the findings of this study are available from the corresponding author upon reasonable request.

## Supplementary Materials

The following supporting information can be downloaded at: https://www.mdpi.com/article/10.3390/toxics13090795/s1, Figure S1: Adsorption (a–c) and desorption (d–f) kinetics fitting curves; Figure S2: Isothermal adsorption experiment fitting curves; Figure S3: SEM images of the surface microstructures of soil (a), PBAT (b), and PLA (c); Figure S4: XPS fine spectra of soil and two types of DMPs; Table S1: Basic physical and chemical properties of two DMPs; Table S2: Physicochemical properties of the tested soil Table S3: Partition coefficient (K_d_ (L/g)) of Cu^2+^ between different soil samples and solutions; Table S4: Separation factors of different soil samples; Table S5: Ion exchange during adsorption equilibrium; Table S6: The pH_PZC_ of soil and two DMPs; Table S7: BET characterization of soil and two DMPs.

## Abbreviations

The following abbreviations are used in this manuscript:

BET	Bet surface area and porosity	PE	Polyethylene
DMPs	Degradable microplastics	PFO	Pseudo-first order
D-R	Dubinin–Radushkevich	PHA	Polyhydroxyalkanoates
FT-IR	Fourier transform infrared	pH_pzc_	Point of zero charge
ICP-OES	Inductively coupled plasma-optical emission spectrometry	PLA	Polylactic acid
ID	Intraparticle diffusion	PP	Polypropylene
LDPE	Low-density polyethylene	PS	Polystyrene
MPs	Microplastics	PSO	Pseudo-second order
NDMPs	Non-degradable microplastics	PVC	Polyvinyl chloride
PBAT	Poly (butylene adipate-co-terephthalate)	SEM	Scanning electron microscopy
PBS	Poly (butylene succinate)	XPS	X-ray photoelectron spectroscopy
