# Supplementary material for: Insights into How Degradable Microplastics Enhance Cu2+ Mobility in Soil Through Interfacial Interaction
Source: Toxics. 2025 Sep 18;13(9):795. doi: 10.3390/toxics13090795 (PMC12474448; doi:10.3390/toxics13090795)
Supplement: Supplementary file 1 [file toxics-13-00795-s001.zip › toxics-3835803-supplementary.pdf]

Supplementary Materials

Insights Into How Degradable Microplastics Enhance Cu^{2+} Mobility in Soil Through Interfacial Interaction

Hongjia Peng [1,2], Bolun Yu [1,2], Zuhong Lin [1,2] and Haipu Li [1,2,*]

1 Center for Environment and Water Resources, College of Chemistry and Chemical Engineering, Central South University, Changsha 410083, China; 18528037456@163.com (H.P.); 222311089@csu.edu.cn (B.Y.); lzhzzzzgkbs@163.com (Z.L.)

2 Key Laboratory of Hunan Province for Water Environment and Agriculture Product Safety, Changsha 410083, China

* Correspondence: lihaipu@csu.edu.cn

Summary

Number of pages: 13

Number of tables: 7

Number of figures: 4

Table Captions

Table S1. Basic physical and chemical properties of two DMPs.
Table S2. Physicochemical properties of the tested soil.
Table S3. Partition coefficient (Kd (L/g)) of Cu^{2+} between different soil samples and solutions.
Table S4. Separation factors of different soil samples.
Table S5. Ion exchange during adsorption equilibrium.
Table S6. The pH_{PZC} of soil and two DMPs.
Table S7. BET characterization of soil and two DMPs.

Figure Captions

Figure S1. Adsorption (a-c) and desorption (d-f) kinetics fitting curves.
Figure S2. Isothermal adsorption experiment fitting curves.
Figure S3. SEM images of the surface microstructures of soil (a), PBAT (b), and PLA (c).
Figure S4. XPS fine spectra of soil (a,b), PBAT (c,d), and PLA (e,f).

Table S1. Basic physical and chemical properties of two DMPs.

Type	Raw and processed materials	Shape	Particle size	Density	Melting point (°C)
PBAT	Petrochemical based	Powder	< 250um	1.22	120
PLA	Biological based	Powder	< 250um	1.24	176

Table S2. Physicochemical properties of the tested soil.

Soil type	pH	Organic matter (g/kg)	Cation exchange capacity (cmol/kg)	Total Cu (mg/kg)
Red soil	4.20 ± 0.01	8.17 ± 0.16	4.89 ± 0.12	21.72 ± 0.66

Table S3. Partition coefficient (Kd (L/g)) of Cu^{2+} between different soil samples and solutions.

Nominal concentration of Cu^{2+} (mg/kg)	Soil	Soil (1% PBAT)	Soil (1% PLA)
100	8.2×10^{-3}	7.3×10^{-3}	7.0×10^{-3}
150	6.5×10^{-3}	6.2×10^{-3}	6.0×10^{-3}
200	4.9×10^{-3}	4.6×10^{-3}	4.6×10^{-3}
250	4.4×10^{-3}	4.0×10^{-3}	3.9×10^{-3}
300	4.1×10^{-3}	3.8×10^{-3}	3.7×10^{-3}

Table S4. Separation factors of different soil samples.

**Soil type**	**Soil**	**Soil (1% PBAT)**	**Soil (1% PLA)**
$R_{L\ Max}$	0.464	0.472	0.475
$R_{L\ Min}$	0.234	0.241	0.242

Table S5. Ion exchange during adsorption equilibrium.

Soil type	Soil	Soil (1% PBAT)	Soil (1% PLA)
D_e (Cu^{2+})	36.53 ± 0.10	35.13 ± 0.31	34.81 ± 0.24
D_e (Mg^{2+})	0.21 ± 0.00	0.17 ± 0.01	0.17 ± 0.00
D_e (Al^{3+})	6.37 ± 0.04	6.30 ± 0.05	6.24 ± 0.08
D_e (K^{+})	1.04 ± 0.06	0.81 ± 0.03	0.82 ± 0.02
D_e (Ca^{2+})	1.59 ± 0.05	1.60 ± 0.05	1.64 ± 0.07
D_e (Mn^{2+})	0.33 ± 0.01	0.27 ± 0.02	0.27 ± 0.01
D_e (H^{+})	0.14 ± 0.01	0.13 ± 0.00	0.13 ± 0.00
P_{ie}	33.6%	34.0%	34.3%

Table S6. The pH_{PZC} of soil and two DMPs.

Samples	Soil	PBAT	PLA
pH_{PZC}	2.49	4.30	3.38

Table S7. BET characterization of soil and two DMPs.

Samples	Soil	PBAT	PLA
Surface Area (m^2/g)	16.918	0.235	0.370
Pore Volume (cm^3/g)	0.0682	0.0009	0.0008
Pore Size (nm)	16.116	15.314	8.825

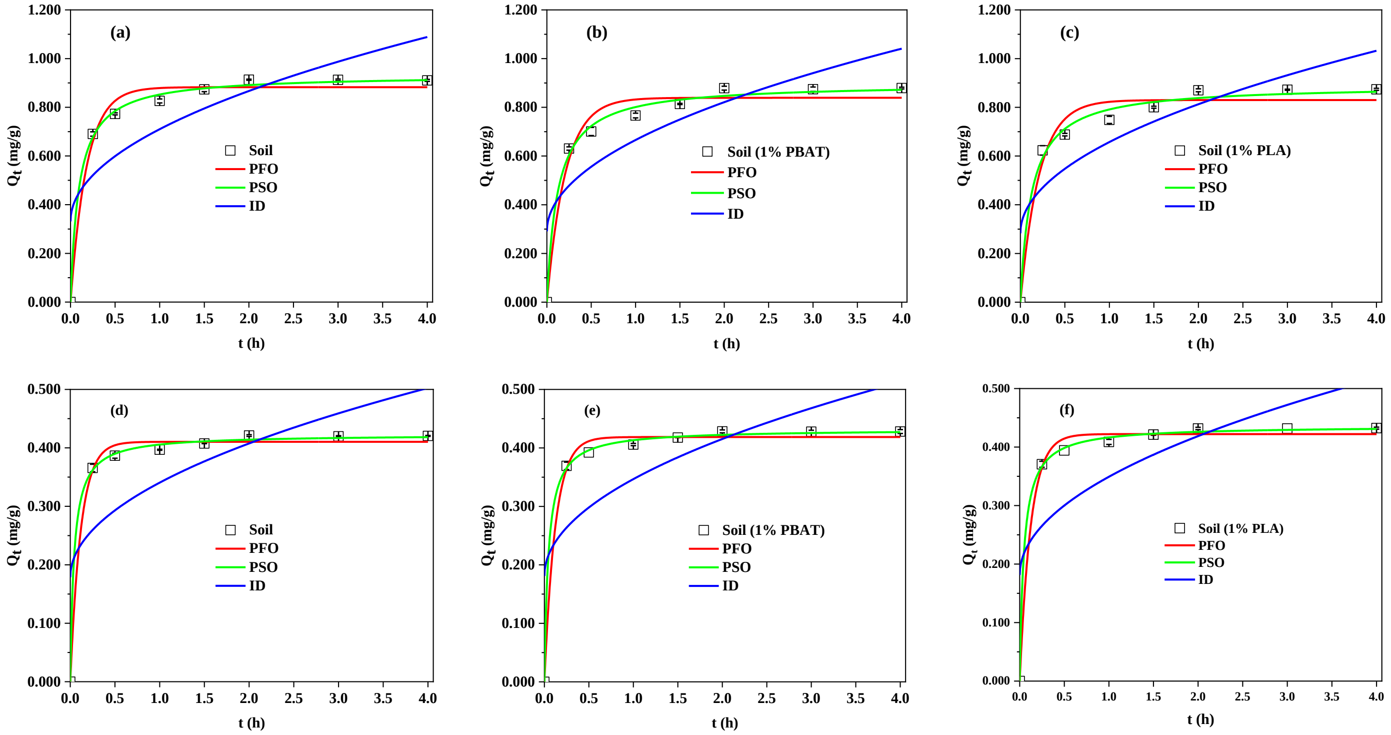

Figure S1. Adsorption (a-c) and desorption (d-f) kinetics fitting curves.

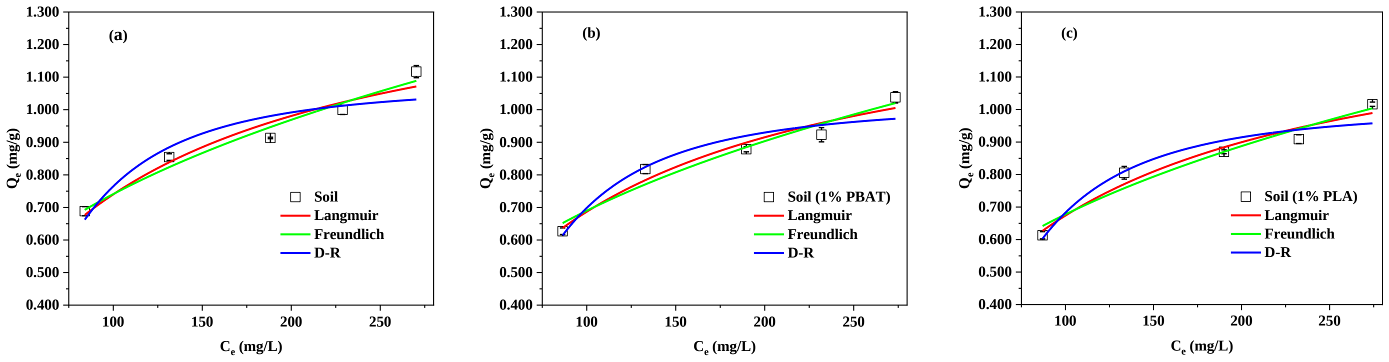

Figure S2. Isothermal adsorption experiment fitting curves.

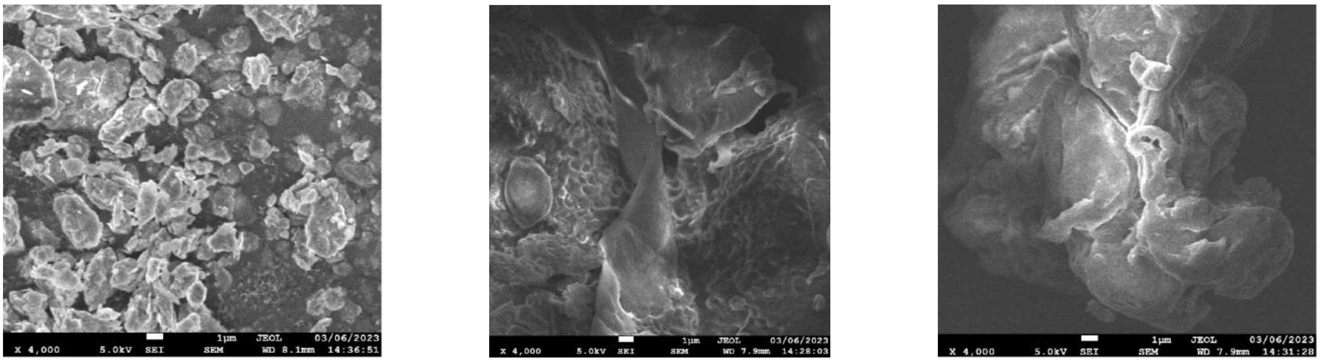

Soil (a) PBAT (b) PLA (c)

Figure S3. SEM images of the surface microstructures of soil (a), PBAT (b), and PLA (c).

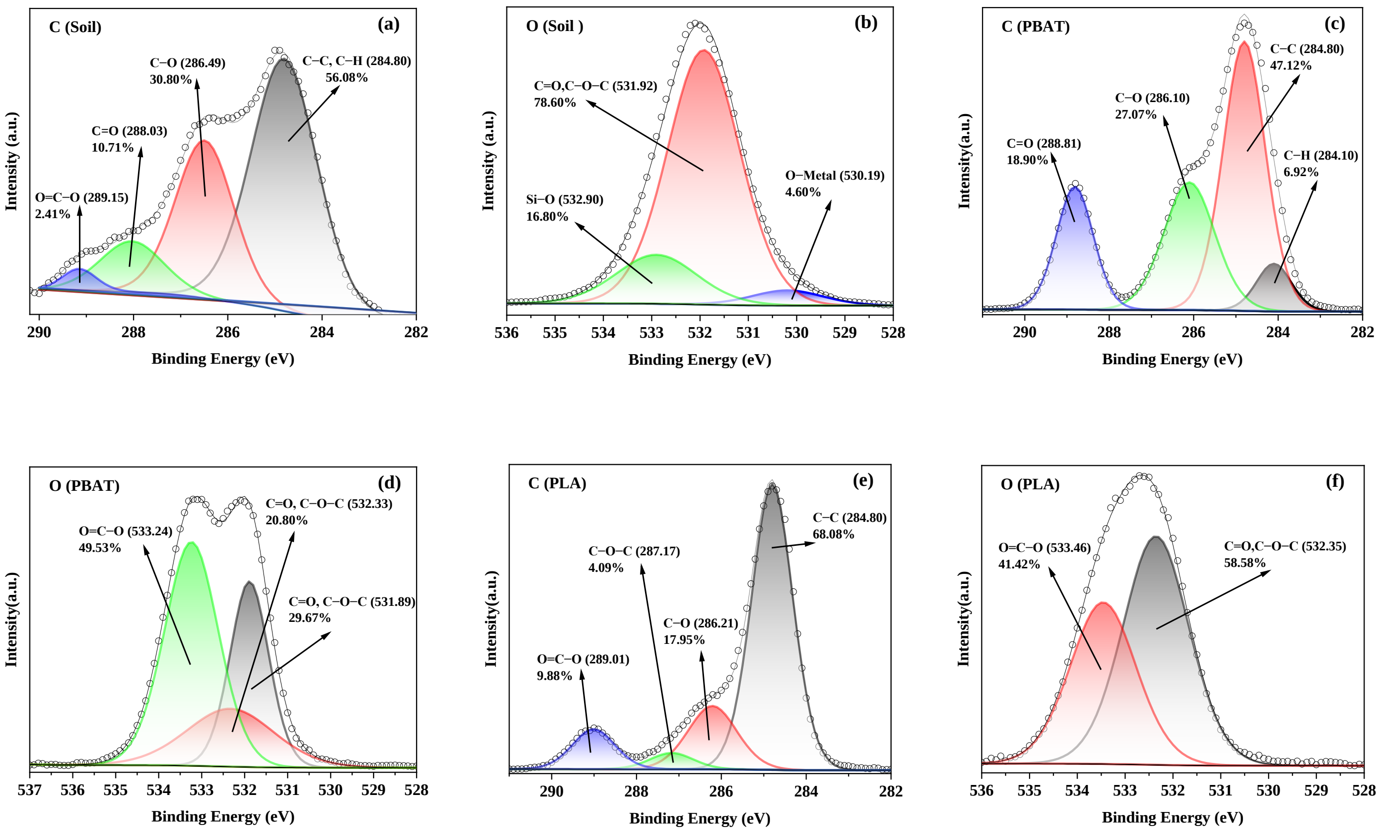

Figure S4. XPS fine spectra of soil (a,b), PBAT (c,d), and PLA (e,f).
